# Association of the long non-coding RNA MALAT1 with the polycomb repressive complex pathway in T and NK cell lymphoma

**DOI:** 10.18632/oncotarget.15453

**Published:** 2017-02-17

**Authors:** Soo Hee Kim, Se Hoon Kim, Woo Ick Yang, Soo Jeong Kim, Sun Och Yoon

**Affiliations:** ^1^ Department of Pathology, Yonsei University College of Medicine, Seoul, Korea; ^2^ Anatomic Pathology Reference Lab, Seegene Medical Foundation, Seoul, Korea; ^3^ Department of Internal Medicine, Yonsei University College of Medicine, Seoul, Korea

**Keywords:** T and NK cell lymphomas, long non-coding RNA, MALAT1, polycomb repressive complex

## Abstract

Recently, various long non-coding RNAs (lncRNAs) have been reported to have significant therapeutic or prognostic value. However, the expression of lncRNAs has not been investigated in T and NK cell lymphoma. Thus, we evaluated the biological and prognostic role of lncRNAs related to the polycomb repressive complex (PRC) and PRC markers in tissue samples and cell lines of T and NK cell lymphoma. Among the tested lncRNAs, MALAT1 was most highly expressed in clinical samples and cell lines. High expression of MALAT1 as well as BMI1 was related to poor prognosis in patients with mature T cell lymphoma. In the tissue samples, BMI1 expression showed a positive correlation with EZH2, SUZ12, H3K27me3, and MALAT1. Multiple linear regression analysis showed that BMI1 expression was independently associated with H3K27me3. Direct binding of MALAT1 to the PRC2 components (EZH2 and SUZ12) was observed in a T cell lymphoma cell line; however, no direct binding of MALAT1 with H3K27me3 and BMI1 (a PRC1 component) was observed.

In T and NK cell lymphomas, MALAT1 was related to poor prognosis. MALAT1 directly binds to EZH2 and SUZ12, and BMI1 activation may be induced possibly through H3K27me3.

## INTRODUCTION

The incidence of T and natural killer (NK) cell lymphoma is particularly high in the East Asian population; however, therapeutic options are currently limited to conventional chemotherapy and radiotherapy with no available target agents [1]. Long non-coding RNAs (lncRNAs) are non-protein-coding RNA molecules longer than 200 nucleotides that regulate gene expression in chromosome remodeling, transcription, and post-transcriptional processes [2]. Recently, the aberrant expression of lncRNAs has been investigated in many human diseases, including various malignant tumors [2–7]. LncRNAs are deregulated in a number of cancers, demonstrating both oncogenic and tumor-suppressive roles, thus suggesting that their aberrant expression may be a substantial contributor to cancer development. Moreover, many studies have demonstrated the clinicopathologic significance of lncRNAs in terms of associations with prognosis or a therapeutic benefit, suggesting possible therapeutic targets or candidate biomarkers[8–10]. With respect to hematologic malignancies, the role of lncRNAs has been studied in B cell lymphoma/leukemia [11–15], but not in T and NK cell lymphoma.

Some lncRNAs are known to play roles in the regulation of gene expression via a mechanism involving interaction with the polycomb repressive complex (PRC) pathway [16]. The PRC proteins consist of two main families, PRC1 (including BMI1) and PRC2 (including EZH2, SUZ12, and EED1) [17]. In general, PRC2 is related to the trimethylation of histone H3 at lysine 27 (H3K27me3), whereas PRC1 interacts in the genome regions via H3K27me3 [18].

Association of lncRNAs and PRC-related markers has been reported in different types of malignant tumors [19–23]. For example, increased expression of the lncRNA metastasis-associated lung adenocarcinoma transcript 1 (MALAT1) in renal cell carcinoma was reported to promote aggressive biological behavior through interactions with EZH2 [24]. In our previous study, a high frequency of patients with T and NK cell lymphomas showed activation of PRC pathway-related markers (EZH2, SUZ12, H3K27me3, and BMI1), and high expression of BMI1 was related to poor prognosis. These results implied that activation of the PRC pathway, especially BMI1 activation, which is induced by PRC2-H3K27me3 activation, may be related to the clinically aggressive behaviors of T and NK cell lymphomas [25]. However, the regulation of lncRNAs and the PRC pathway as well as the prognostic role of lncRNAs in T and NK cell lymphomas remain unclear.

Therefore, in the present study, we investigated the expression of lncRNAs in T and NK cell lymphomas and analyzed the relationships between these lncRNAs and the PRC pathway as well as their prognostic implications.

## RESULTS

### Expression of PRC markers determined by immunohistochemistry in T and NK cell lymphoma tissue

**Figure 1:**
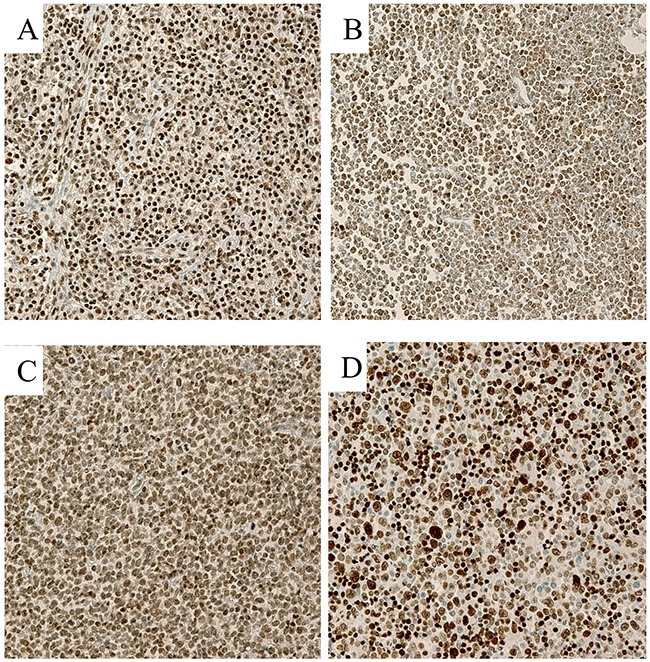
Representative immunohistochemical stains in T cell lymphoma samples (400×) **(A)** EZH2, **(B)** SUZ12, **(C)** H3K27me3, and **(D)** BMI1 Lymphoma cells showed strong nuclear expression of each marker.

As shown in Figure 1, many lymphoma cases showed high expression of EZH2, SUZ12, H3K27me3, and BMI1 in the tumor nuclei. The frequencies of cases showing high expression, which was defined as positive expression in>75% of tumor cells, were 55.2% (91/167) for EZH2, 32.3% (54/167) for SUZ12, 37.1% (62/167) for H3K27me3, and 31.1% (52/167) for BMI1.

### Expression of lncRNAs in T and NK cell lymphoma tissues and cell lines

**Figure 2:**
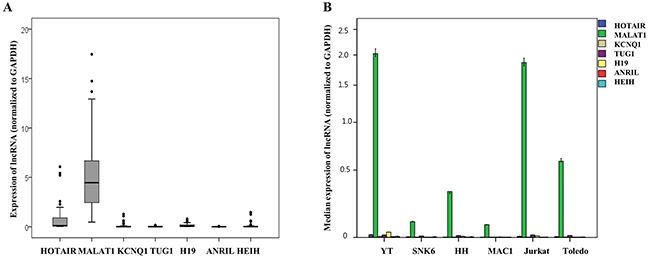
Quantitative expression analysis of seven long non-coding RNAs (lncRNAs) mRNA normalized to GAPDH expression in (A) clinical samples of patients with T and NK cell lymphomas and (B) six cell lines **(A)** MALAT1 showed distinctively higher expression when compared to the other types of lncRNAs in clinical samples of T and NK cell lymphomas. **(B)** MALAT1 showed distinctively higher expression when compared to other types of lncRNAs in cell lines derived from various types of T and NK cell lymphomas and a cell line derived from a patient with diffuse large B-cell lymphoma (Toledo).

Among the seven types of lncRNAs tested, HOTAIR, MALAT1, KCNQ1, TUG1, H19, ANRIL, and HEIH, in the clinical samples of patients with T and NK cell lymphomas, MALAT1 showed distinctively high expression when compared to the others, followed by HOTAIR. The expression of the other lncRNAs, KCNQ1, TUG1, H19, ANRIL, and HEIH, was much lower or quite subtle (Figure 2A and Supplementary Table 1).

MALAT1 also showed distinctively high expression when compared to the other lncRNAs tested in cell lines derived from patients with various types of T and NK cell lymphomas and a cell line derived from a patient with diffuse large B-cell lymphoma (Figure 2B).

### MALAT1 expression and PRC activation in T and NK cell lymphoma tissues

**Figure 3:**
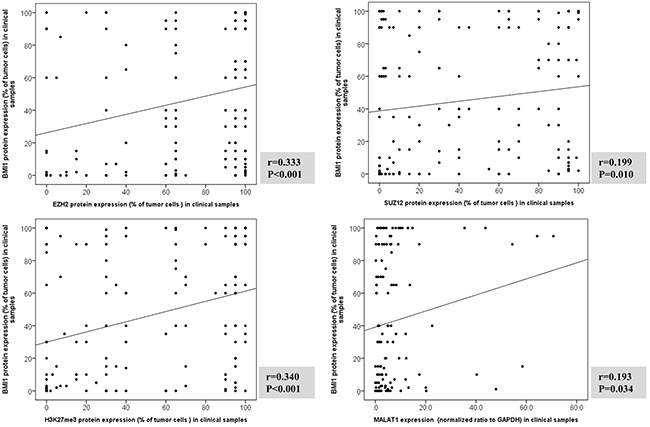
Correlation analysis between BMI1 protein expression and EZH2, SUZ12, H3K27me3, and MALAT1 in clinical samples of T and NK cell lymphomas The expressions of BMI1, EZH2, SUZ12 and H3K27me3 evaluated protein by immunohistochemistry. MALAT1 expression measured using real-time RT-PCR. Correlation between BMI1 protein expression and EZH2, SUZ12 and H3K27me3, and MALAT1 mRNA was examined with two-sided Spearman coefficient. MALAT1 was positively correlated with BMI1 but not with EZH2, SUZ12, or H3K27me3, whereas BMI1 was positively correlated with EZH2, SUZ12, or H3K27me3.

The expression levels of MALAT1 and the PRC-related markers EZH2, SUZ12, H3K27me3, and BMI1 showed positive correlations with each other. In particular, MALAT1 expression was positively correlated with BMI1 but not with EZH2, SUZ12, or H3K27me3, whereas BMI1 was positively correlated with all other markers (Figure 3 and Supplementary Figure 1). In multiple linear regression analysis, H3K27me3 was independently associated with BMI1 expression, and MALAT1 also revealed a tendency to be associated with BMI1 expression (Table 1).

**Table 1:** Multiple linear regression analysis of markers influencing BMI1 expression in clinical samples of patients with T and NK cell lymphoma

	ß (95% CI)	t	P value
EZH2	0.012 (-018 to 0.21)	0.136	0.892
SUZ12	0.155 (-0.02 to 0.34)	1.816	0.072
H3K27me3	0.383 (0.21 to 0.56)	4.332	<0.001
MALAT1	0.165 (-0.02 to1.07)	1.913	0.058

### Associations of MALAT1 expression and PRC activation with patient clinical characteristics

No significant difference in MALAT1 expression was noted among patients classified into the different subtypes of T and NK lymphomas: extranodal NK/T cell lymphoma of nasal type (ENKTL); mature (peripheral) T cell lymphoma(PTCL), including PTCL-not otherwise specified (NOS), angioimmunoblastic T cell lymphoma (AITL), and anaplastic large cell lymphoma (ALCL); and precursor T lymphoblastic lymphoma/leukemia (T-LBL) (Supplementary Figure 2). In addition, no distinct association was noted between MALAT1 expression and clinical factors such as age, pretreatment lactate dehydrogenase level, Ann-Arbor stage, International Prognostic Index score, anatomical sites of the tumor, or bone marrow involvement (Table 2).

**Table 2:** Correlation between MALAT1 expression and clinicopathologic variables

	Low MALAT1 expression (%)	High MALAT1 expression (%)	P value
Subtype			0.872
ENKTL	11(52.4)	10(47.6)	
PTCL, NOS	15(60.0)	10(40.0)	
AITL	7(53.8)	6(46.2)	
ALCL	16(66.7)	8(33.8)	
T-LBL	7(53.8)	6(46.2)	
Sex			0.250
Male	31(62.0)	19(38.0)	
Female	14(48.3)	15(51.7)	
Age (years)			1.000
<60	30(56.6)	23(43.4)	
≥60	15(57.7)	11(42.3)	
Primary site of tumor			0.394
Head and neck	7(53.8)	6(46.2)	
Lymph node	27(55.1)	22(44.9)	
Gastrointestinal tract	5(71.4)	2(28.6)	
Soft tissue and bone	5(83.3)	1(16.7)	
Others (solid organs)	1(25.0)	3(75.0)	
Ann Arbor stage			0.219
Stage I & II	11(78.6)	3(21.4)	
Stage III&IV	27(58.7)	19(41.3)	
LDH level			1.000
Normal	12(66.7)	6(33.3)	
Elevated	25(69.4)	11(30.6)	
Bone marrow involvement			0.397
Absent	28(63.6)	16(36.4)	
Present	9(50.0)	9(50.0)	
IPI score			0.165
0-2	26(72.2)	10(27.8)	
3-5	12(52.2)	11(47.8)	

Abbreviations: LDH, lactate dehydrogenase; IPI, International Prognostic Index; ENKTL, extranodal natural killer/T-cell lymphoma; PTCL-NOS, peripheral T cell lymphoma, not otherwise specified; AITL, angioimmunoblastic T cell lymphoma; T-LBL, T lymphoblastic leukemia/lymphoma.

Information was not available in some cases; the actual number of cases (percentage) is presented in the parentheses.

**Figure 4:**
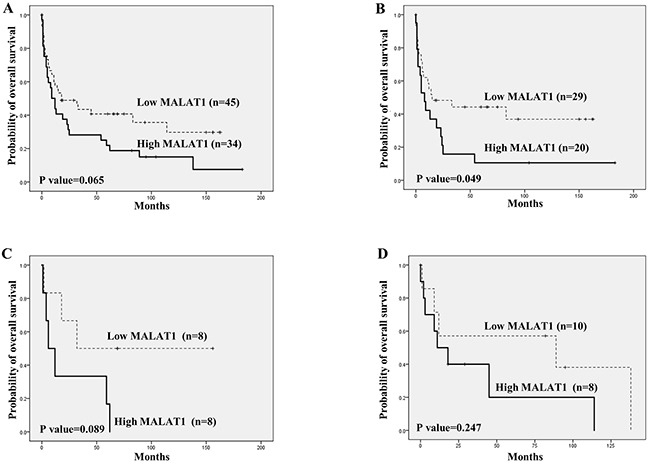
Overall survival analysis according to MALAT1 expression in T and NK cell lymphomas using Kaplan-Meier curves **(A)** Overall T and NK cell lymphoma cases. **(B)** Mature (peripheral) T cell lymphoma. **(C)** Precursor T lymphoblastic lymphoma/leukemia (T-LBL). **(D)** Extranodal NK/T cell lymphoma of nasal type (ENKTL). High MALAT1 expression showed a tendency to be related to lower overall survival in all T and NK cell lymphoma cases **(A)**. In the cases of mature (peripheral) T cell lymphoma, high MALAT1 expression was significantly related to a lower overall survival rate **(B)**, and cases of T-LBL showed a similar tendency **(C)**. For ENKTL, no distinct finding between MALAT1 expression and overall survival was noted **(D)**.

However, high MALAT1 expression tended to be related to lower overall survival (Figure 4A). In particular, high MALAT1 expression was significantly related to a lower overall survival rate in cases of mature PTCL and T-LBL (Figure 3B and 3C). However, no such relationship between MALAT1 expression and overall survival was noted for patients with ENKTL (Figure 3D).

Similar to our previous study [25], high BMI1expression was significantly associated with lower overall survival in the mature T cell lymphoma group (P<0.001) as well as among all T and NK cell lymphoma cases (P<0.001). No significant association between BMI1 and overall survival rate was observed in cases of T-LBL (P=0.237), and marginal significance was noted (P=0.053) in cases of ENKTL (Supplementary Figure 3).

### Correlation between MALAT1 and PRC-related markers in the HH cell line

**Figure 5:**
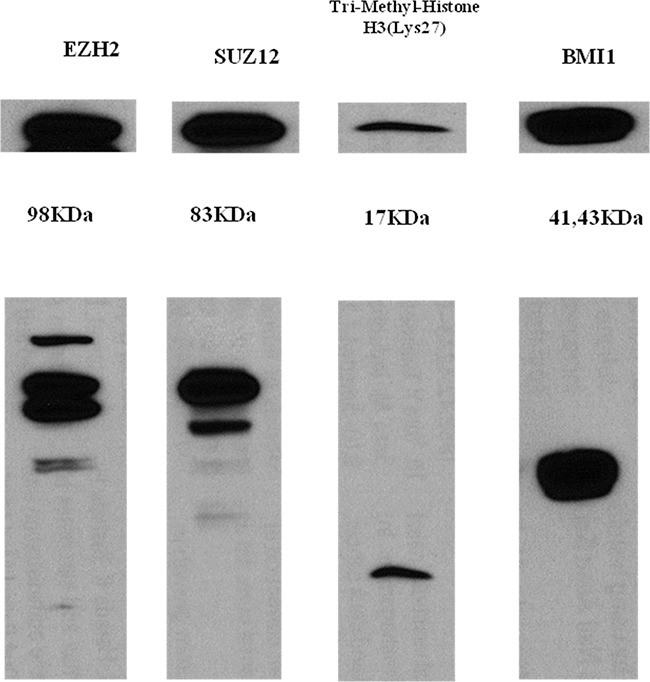
Western blot analysis of EZH2, SUZ12, H3K27me3, and BMI-1 in the HH cell line All PRC pathway markers showed strong baseline expression in the non-treated HH cell line.

**Figure 6:**
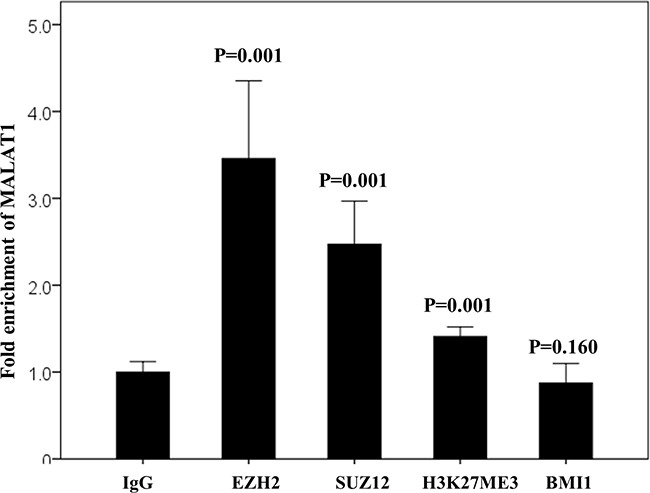
RNA immunoprecipitation assay in the non-treated HH cell line MALAT1 directly binds with EZH2, SUZ12, and H3K27me3 but not with BMI1. IgG antibody was used as the control.

In the non-treated cell line derived from a patient with T cell lymphoma (HH cells), baseline protein expression levels of PRC-related markers, especially EZH2, SUZ12, BMI1, and H3K27me3, were very strong (Figure 5). To investigate the potential binding of MALAT1 with these PRC-related markers, an RNA immunoprecipitation study was performed. In the non-treated HH cell lines, MALAT1 was significantly more enriched with the antibodies of EZH2, SUZ12, and H3K27me3 when compared to the control antibody IgG. However, no significant binding of MALAT1 with the BMI1 antibody was observed (Figure 6).

### Changes of PRC-related markers following MALAT1 inhibition in the HH cell line

**Figure 7:**
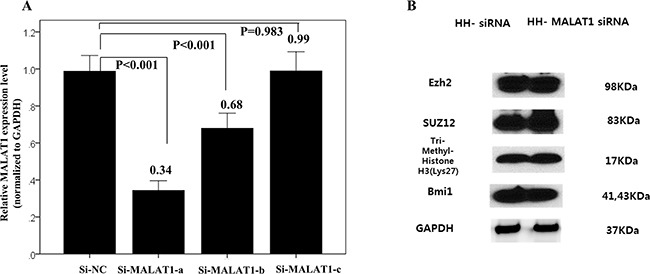
Changes of interaction with PRC-related markers according to MALAT1 inhibition **(A)** Transfected oligomers of si-MALAT1, especially, si-MALT1-a, efficiently inhibited MALAT1 expression in the HH cell line. **(B)** Western blot analysis of four PRC pathway markers after 48h transfection of si-MALAT1 in the HH cell line. After 48h of transfection, the expression of PRC-related markers, EZH2, SUZ12, H3K27me3, and BMI1 were not significantly decreased.

We confirmed that the transfected oligomers of the small interfering RNAs (si-RNAs) specific to MALAT1 (si-MALAT1), especiallysi-MALT1-a, efficiently inhibited MALAT1 expression (Figure 7A). At 48h after transfection, expression of the PRC-related markers EZH2, SUZ12, H3K27me3, and BMI1 was not significantly decreased according to the results of western blot analysis (Figure 7B).

**Figure 8:**
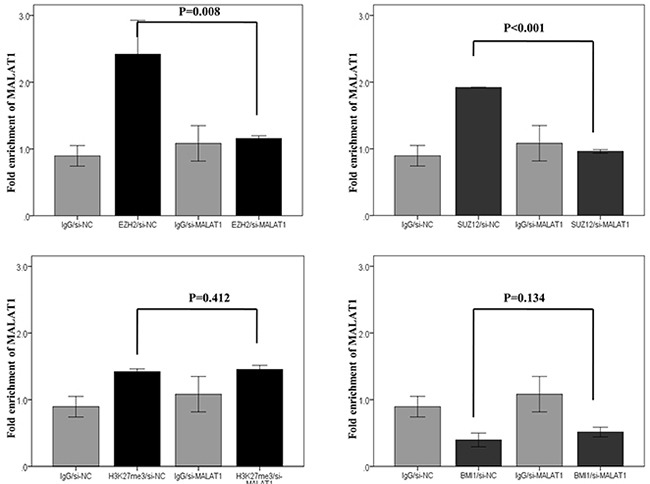
RNA immunoprecipitation of four PRC pathway markers after MALAT1 knockdown in HH cell lines Interactions of MALAT1 with antibodies to EZH2 and SUZ12 significantly decreased after transfection of si-MALAT1 when compared to those observed after transfection of scramble siRNA. Interactions of MALAT1 with the antibody of H3K27me3 and BMI1 did not significantly change even after MALAT1 inhibition.

In RNA immunoprecipitation, the fold enrichment level of MALAT1 bound to the control (IgG) did not change, even after knockdown of MALAT1. This result excluded the possibility of the non-specific binding of MALAT1 to the negative control or other non-target proteins. The fold enrichment levels of MALAT1 bound to EZH2 and SUZ12significantly decreased after transfection of si-MALAT1 when compared to those of cells transfected with scramble siRNA. However, the fold enrichment levels of MALAT1 bound to H3K27me3 and BMI1 were not significantly changed, even after MALAT1 inhibition. The fold enrichment levels of MALAT1 bound to EZH2, SUZ12, and H3K27me3 were similar (Figure 8).

## DISCUSSION

Our study shows that MALAT1 expression is increased more substantially than the other lncRNAs evaluated in T and NK cell lymphomas. The MALAT1 locus at 11q13.1 has been identified to harbor chromosomal translocation break points associated with cancer [2, 7, 26]. MALAT1 is also known as nuclear-enriched abundant transcript2 (NEAT2), and was first identified as a novel non-coding RNA in non-small cell lung cancer [27]. Recently, the role of MALAT1 in tumor development has been extensively reported [19, 21, 24, 26, 28–33]. MALAT1 was observed to promote malignancy in osteosarcoma, as its knockdown inhibited cell proliferation and invasion, and suppressed metastasis [34–36]. Silencing of MALAT1 expression reduced cell proliferation and invasion and increased apoptosis in renal cell carcinoma cells [24]. In addition, recent reports have indicated that MALAT1 expression is significantly associated with poor prognosis in various malignant tumors [28, 29, 31, 37, 38]. The results of the present study also demonstrated a worse prognosis for T and NK lymphoma patients in the high MALAT1 expression group, especially for those in the mature PTCL group, including PTCL-NOS, AITL and ALCL.

A recent study showed that the MALAT1 expression level was upregulated and promoted the activity of the PRC2 complex by interacting with the SUZ12 protein in bladder cancer [39]. In chemotherapy-resistant prostate cancer, MALAT1 was shown to directly interact with EZH2 protein, suggesting that MALAT1 may play a key role as an RNA cofactor of EZH2 and that the EZH2-MALAT1 association may be a new therapeutic target [21].

In our previous study, frequent activation of PRC pathway-related markers (EZH2, SUZ12, H3K27me3, and BMI1) was observed in patients with T and NK cell lymphomas, and the expression levels of these marker were closely correlated. Moreover, high expression of BMI1 was related to the poor prognosis of T and NK cell lymphoma patients. Therefore, the results of this previous study implied that activation of the PRC pathway, especially BMI1 activation, which is induced by PRC2-H3K27me3 activation, may be related to the clinically aggressive behaviors of T and NK cell lymphomas [25].

In the present study, MALAT1, as well as the PRC-related markersEZH2, SUZ12, H3K27me3, and BMI1, were found to be positively correlated. Notably, H3K27me3 was identified as an independent factor related to BMI1 activation. To assess the direct binding between MALAT1 and PRC-related pathway markers, an RNA immunoprecipitation study was performed using the T cell lymphoma cell line, which shows high endogenous expression of PRC-related markers; this assay confirmed the direct binding of MALAT1 to EZH2, SUZ12, and H3K27me3. The bound MALAT1 level decreased in the following order: EZH2>SUZ12> H3K27me3; however, MALAT1 did not directly bind to BMI1. To exclude the possibility that the binding of MALAT1 to PRC-related markers may be an HH cell line-specific phenomenon, the MALAT1expression level was modified using an siRNA transfection method. When MALAT1 expression was knocked down, the level of binding of MALAT1 to EZH2 and SUZ12 significantly decreased, whereas the binding to H3K27me3 or BMI1 was not affected.

Collectively, these observations suggest a molecular mechanism contributing to the development of T cell lymphomas. During the sequential processes of PRC pathway activation and chromatin remodeling in T cell lymphomas, MALAT1 may directly and specifically bind to PRC pathway proteins, particularly EZH2 and SUZ12, members of the PRC2 family, which may in turn induce H3K27me3. The low level of binding of H3K27me3 to MALAT1 may be an indirect result of the sequential histone methylation through PRC recruitment. Because the protein expression of EZH2 or SUZ12 was not changed, even after the MALAT1 expression level was decreased by siRNA transfection, the low or baseline level of MALAT1 bound to H3K27me3 seemed to be unchanged due to the intact action of EZH2 and SUZ12. In fact, the fold enrichment level was similar for MALAT1 bound to EZH2, SUZ12, and H3K27me3 after knockdown of MALAT1. BMI1, a member of the PRC1 family, may be specifically and directly induced in response to H3K27me3; therefore, the correlation of BMI1 expression with the expression of PRC2 markers and MALAT1 could be an indirect consequence of the sequential processes of PRC pathway activation and chromatin remodeling in T cell lymphoma, without direct binding between BMI1 and MALAT1.

**Figure 9:**
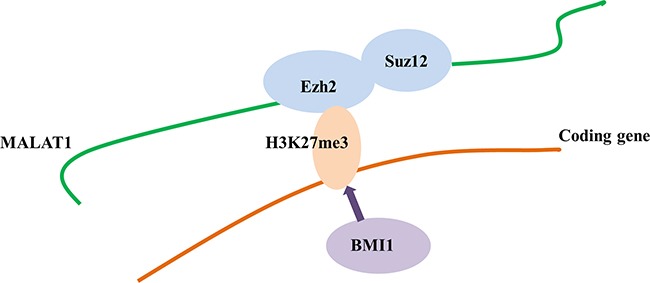
Hypothetical mechanism of the interaction of MALAT1 and the PRC repressive pathway MALAT1 may directly and specifically bind to EZH2 and SUZ12. H3K27me3 may occur sequentially through EZH2 and SUZ12 recruitment by MALAT1, which may in turn induce BMI1 activation. Therefore, although H3K27me3 and BMI1 do not bind directly to MALAT1, their activation is induced by MALAT1 via recruitment of EZH2 and SUZ12.

The direct binding of MALAT1 to the PRC2 proteins EZH2, H3K27me3, and SUZ12 has been reported previously [21, 24, 39]. The present study confirmed the direct binding between MALAT1 and the PRC2 subunits EZH2 and SUZ12 in T cell lineage lymphoma. However, direct activation between MALAT1 and BMI1, a subunit of PRC1, was not identified in T cell lineage lymphoma. Nevertheless, our results suggest that MALAT1 may be an important molecule for sustaining PRC2-induced H3K27me3, leading to the subsequent BMI1 activation in T and NK cell lymphomas (Figure 9). The poor prognosis of T cell lymphoma patients with high BMI1 expression suggest that activated BMI1 might be involved in the progression to a high-risk phenotype.

In the present study, MALAT1 knockdown did not suppress the protein expression of EZH2, SUZ12, BMI1, and H3K27me3 in the *in vitro* analysis. Because the endogenous expression level of these molecules was very high, the 48-h transfection time using siRNA oligonucleotides might not have been sufficient to decrease the levels of these molecules, although the functional interaction of MALAT1 and PRC-related proteins was affected during this period. Alternatively, the short duration of the observation period and the lack of quantitative changes of PRC pathway markers might have contributed to the lack of an effect of MALAT1 knockdown on cell survival in the present study. Further studies with longer observation times should be conducted using a small hairpin RNA transfection method or a vector-based system.

In summary, the expression of MALAT1 was increased in T and NK cell lymphoma, and high MALAT1 expression was associated with inferior overall survival, especially for patients with mature T cell lymphoma subtypes. Direct binding of MALAT1 with EZH2 and SUZ12 was verified through RNA immunoprecipitation analysis, and further results suggested that overexpressed MALAT1 may induce BMI1 activation, which is itself related to poor patient prognosis. Collectively, these findings demonstrate that MALAT1 could serve as a prognostic marker as well as a therapeutic target in T and NK cell lymphomas.

## MATERIALs AND METHODS

### Patients and tissue samples

A total of 167 clinical samples obtained from patients with T and NK cell lymphomas diagnosed at the Department of Pathology at Severance Hospital from 1999 to 2013 were included in this study. The inclusion criteria were as follows: 1) available paraffin blocks, 2) confirmed diagnosis by two pathologists (S.H.K and S.O.Y) according to current World Health Organization criteria [1]. Overall, 56 patients were diagnosed with ENKTL, 44 with PTCL-NOS, 16 with AITL, 32 with ALCL (16 anaplastic lymphoma kinase [ALK]-positive and 16 ALK-negative), and 19 with T-LBL. Clinical information was obtained from medical record review, and survival analysis was performed for 135 patients with available clinical data. The clinicopathologic characteristics of patients are summarized in Supplementary Table 2. This study was approved by the Institutional Review Board of Severance Hospital.

### Cell lines and reagents

The following cell lines derived from patients with T or NK cell lymphomas and B cell lymphoma were used in the present study: YT, an Epstein-Barr virus (EBV)-positive human NK-like leukemic cell line; SNK6, derived from an EBV-positive patient with NK/T cell lymphoma; Jurkat, a T lymphoblastic leukemia cell line; HH, a cell line derived from a patient with cutaneous T cell lymphoma; and MAC1, a cell line derived from a patient with ALK-negative ALCL were used in the study. All the cell lines derived from T or NK cell lymphomas were kindly provided by Prof. Jeon YK (Seoul National University College of Medicine, Seoul, Korea). The Toledo cell line, established from a patient with diffuse large B-cell lymphoma, purchased from American Type Culture Collection, was also used in this study for comparison. The reagents used for cell culture are summarized in Supplementary Table 4.

### Tissue microarray construction

Tissue microarray blocks were prepared as described previously [40]. The hematoxylin–eosin slides were reviewed and representative formalin-fixed paraffin-embedded (FFPE) archival blocks were selected for each case. Each block contained non-neoplastic tonsil tissue. Selected core tissue (3 mm in diameter) was obtained from the individual FFPE blocks (donor blocks) and arranged in recipient paraffin blocks (tissue microarray blocks) using a trephine apparatus. All tissue microarray blocks were confirmed to contain proper lymphoma lesions and non-neoplastic tonsils after hematoxylin and eosin staining.

### Immunohistochemistry and assessment

Immunohistochemistry for EZH2, SUZ12, BMI1, and H3K27me3 was performed on the tissue microarray blocks following a standard protocol, using a Ventana automatic immunostainer (Ventana, Benchmark, Tuscan, AZ). The primary antibodies used in this study and the specific immunohistochemistry conditions are listed in Supplementary Table 7. After deparaffinization, heat-induced antigen retrieval was performed using citrate buffer (CC1 protocol; Ventana) of pH 6.0. Reactivity was visualized using the Ultra-View detection kit (Ventana). The positive rate for each marker was scored independently by two pathologists (S.H.K and S.O.Y). EZH2, SUZ12, BMI1, and H3K27me3 all showed nuclear expression. The percentage and intensity of positive-stained tumor cells were recorded by manually counting at least 500 tumor cells from representative fields in each case. The cut-off value for high expression of EZH2, SUZ12, H3K27me3, and BMI1 was 75% of tumor cells showing moderate to strong intensity as described in our previous study [25].

### Analysis for lncRNA expression in clinical samples

FFPE tissue sections were prepared and stained with hematoxylin and eosin, and then the tumor areas were confirmed and marked under the microscope. The marked FFPE areas were dissected at a 10-mm thickness using a microtome. In general, four slices of a tissue section per case were used for RNA extraction. Total RNA was isolated using an RNeasyFFPEKit (Qiagen, Hilden, Germany) according to the manufacturer instructions. Extracts of RNA were verified by measuring the ratios of A260/A280 and A260/A230 with an ND-1000 NanoDropspectrophotometer (NanoDrop, Wilmington, DE, USA). One step of reverse transcription and real-time PCR was performed using a Onestep SYBR PrimeScript RT-PCR kit (Takara, Japan). Primers for amplification of seven lncRNAs, HOTAIR, MALAT1, KCNQ1, TUG1, H19, ANRIL, and HEIH, and the reference gene *GAPDH* are summarized in Supplementary Table 3, which were designed using the Primer3-web interface (http://frodo.wi.mit.edu/primer3/input.htm). Amplification was performed using an ABI StepOnePlus™ system (Applied Biosystems, Foster City, CA, USA). Relative lncRNA expression levels were normalized to the *GAPDH* expression level by the comparative cycle threshold (Ct) method (2^−ΔΔCt^). The experiment was performed three times. After stepwise quality assessment, appropriate cases were selected for statistical analyses to detect differences in the lncRNA expression levels in tissue samples (Supplementary Figure 4).

### Transfection, RNA isolation, and real-time PCR

Knockdown of MALAT1 was performed using three types of si-MALAT1 duplexes, si-MALAT1-a, -b, and -c (Bioneeroligosynthesis, Daejeon, South Korea), and a scramble negative control, si-NC (SN-1002, Bioneeroligosynthesis). The siRNAs were transfected using 4D-Nucleofector X unit (V4XC-1024, Lonza, Basel, Switzerland) and the Lonza 4D Nucleofector™system (Lonza) according to the manufacturer instructions. In brief, cells were grown in 6well plates and transfected with cocktails of the three si-MALAT1s (a, b, and c) at a concentration of 200pmol per well. After checking the transfection efficiency, each si-MALAT1 of a 200pmol concentration was individually transfected to the cells. Sequences of si-MALAT1 are summarized in Supplementary Table 5. The cells were harvested after 48h. Experiments were performed independently three or more times. After initial screening of the transfection efficiency in the T and NK cell lymphoma cell lines, the HH cell line was finally selected for further study.

RNA was isolated with RNeasy plus mini kit (Qiagen), and cDNA was reverse transcribed using a cDNA synthesis kit (Invitrogen, Carlsbad, CA, USA). The TaqMan probes were as follows: GAPDH (Hs99999905-m1, ABI; Life Technologies), MALAT1 (Hs00273907-s1, ABI). Quantitative PCR was performed using ABI StepOnePlus™ (Applied Biosystems) under the following conditions: 50°C for 2 min, 95°C for 10 min, 40 cycles of 95°C for 15sec, and 60°C for 1min. Relative expression levels of MALAT1were determined by the comparative method (2^−ΔΔCt^) against the reference gene *GAPDH*.

### RNA immunoprecipitation assay

RNA immunoprecipitation was performed using Imprint® RNA Immunoprecipitation Kit (RIP, Sigma-Aldrich, St. Louis, MO, USA) according to the manufacturer protocols. From the RNA immunoprecipitation fraction, RNA was purified with TRI Reagent (T9424, Sigma-Aldrich), and then treated with DNase I (AMPD1, Sigma-Aldrich). cDNA was synthesized using the cDNA Synthesis Kit (Invitrogen), and fold enrichment of MALAT1 expression was finally determined through quantitative real-time PCR. MALAT1 fold enrichment of the RNA immunoprecipitation fraction for each target antibody was normalized to the RNA immunoprecipitation fraction of the control antibody (IgG). The details of the antibodies used in this study are summarized in Supplementary Table 6.

### Western blot analysis

The antibodies used are summarized in Supplementary Table 6. For visualization, the proteins were enhanced using Amersham ECL Plus Western Blotting Detection System (GE Healthcare Life Sciences, UK). The protein expression level was determined relative to the GAPDH level.

### Statistical analysis

High or low expression of lncRNAs was determined using Youden's index for survival. Pearson's χ^2^ test or Fisher's exact test was used to compare differences between variables, and the Spearman coefficient was used for correlation analysis. Overall survival curves were plotted using the Kaplan–Meier method and compared using the log-rank test. Multiple linear regression analysis was used for probing the interactions of markers. A P-value <0.05 was considered statistically significant. Statistical analyses were performed using IBM SPSS 22 software for Windows (IBM Corp, Somers, NY, USA).

## SUPPLEMENTARY MATERIALS FIGURES AND TABLES


