# Supplementary material for: Association of the long non-coding RNA MALAT1 with the polycomb repressive complex pathway in T and NK cell lymphoma
Source: Oncotarget. 2017 Feb 17;8(19):31305–17. doi: 10.18632/oncotarget.15453 (PMC5458209; doi:10.18632/oncotarget.15453)
Supplement: Supplementary file 1 [file oncotarget-08-31305-s001.pdf]

Association of the long non-coding RNA MALAT1 with the polycomb repressive complex pathway in T and NK cell lymphoma

SUPPLEMENTARY FIGURES AND TABLES

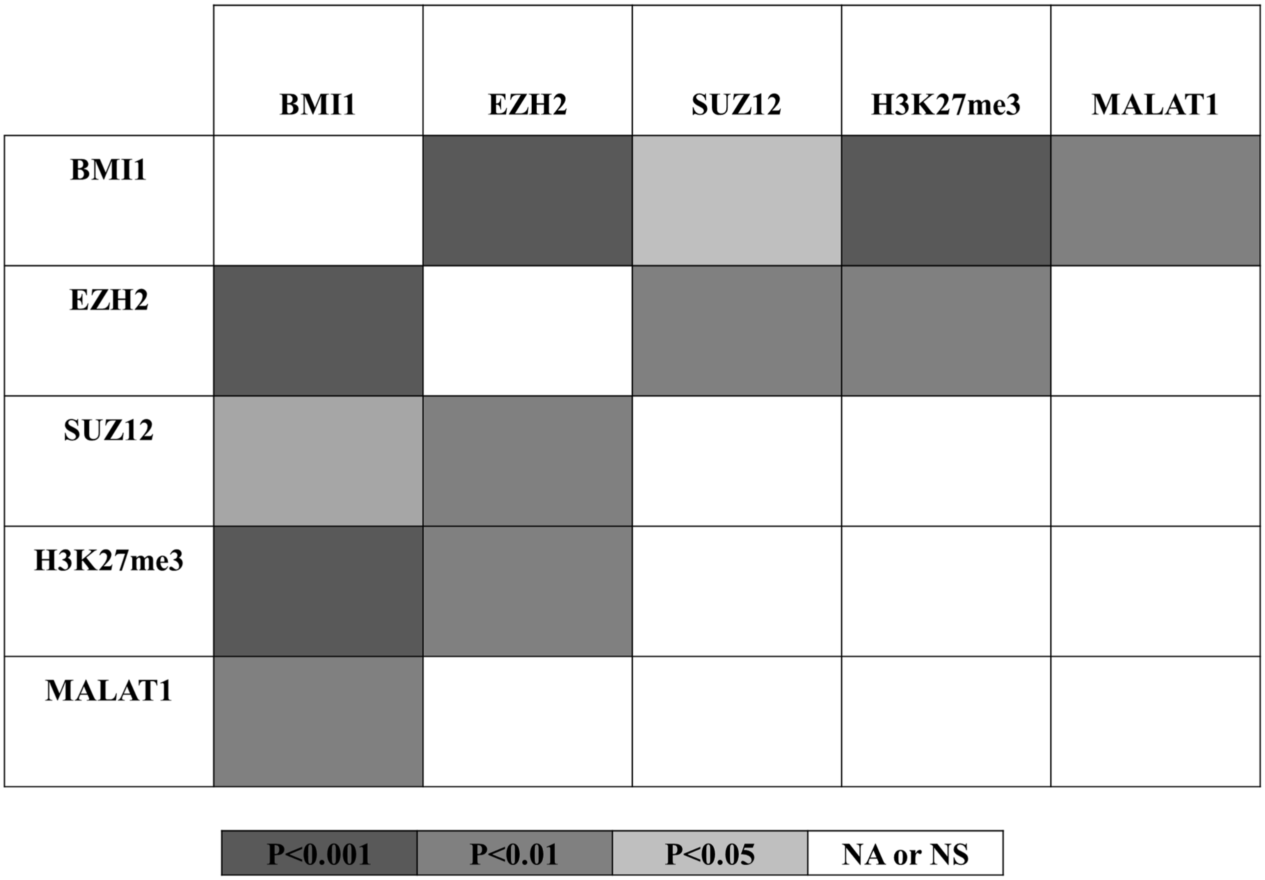

Supplementary Figure 1: BMI1 expression and associations with EZH2, SUZ12, H3K27me3, and MALAT1 expression in clinical samples of patients with T and NK cell lymphoma determined by correlation analysis. MALAT1 was positively correlated with BMI1 but not with EZH2, SUZ12, or H3K27me3, whereas BMI1 was positively correlated with all other markers.

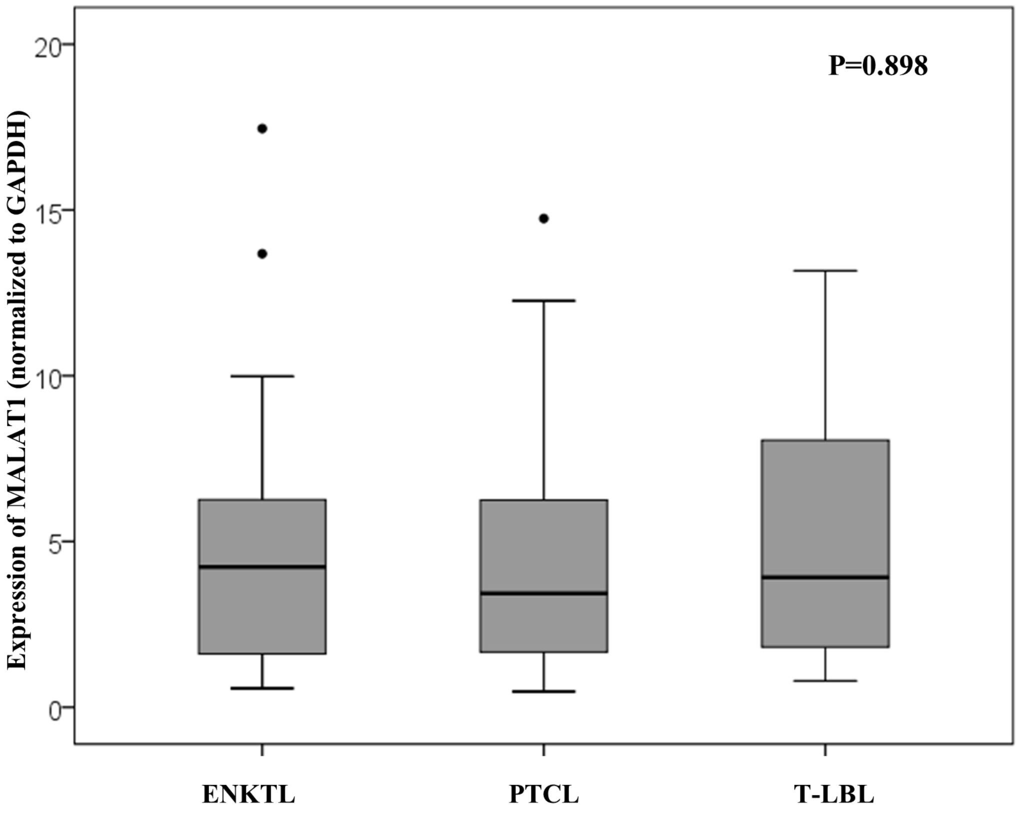

Supplementary Figure 2: Results of quantitative expression analysis of MALAT1 normalized to GAPDH in subtypes of T and NK cell lymphomas. No significant difference of MALAT1 expression was noted according to subtypes. PTCL indicates mature (peripheral) T cell lymphoma, including PTCL-NOS, AITL, and ALCL.

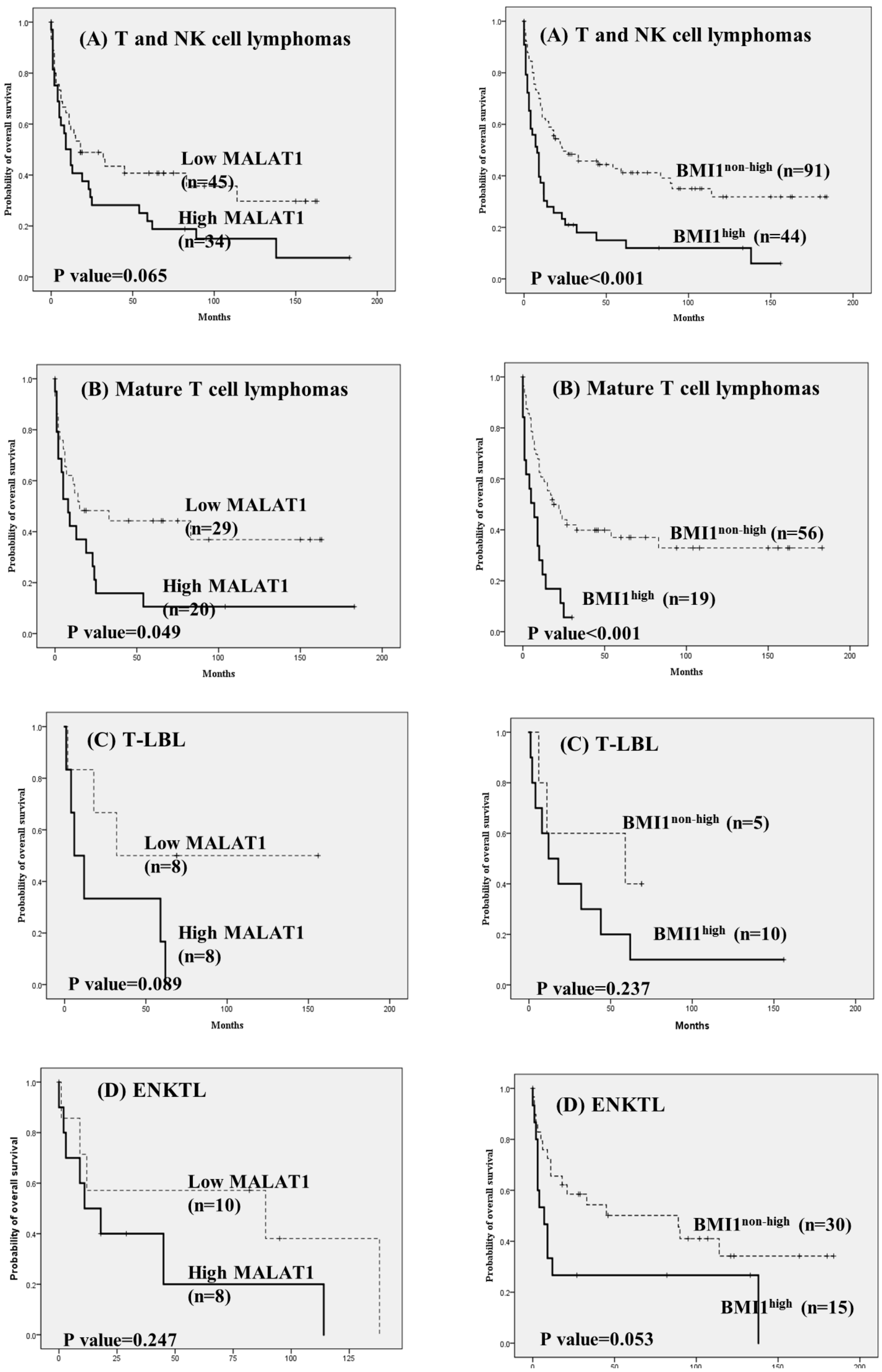

Supplementary Figure 3: Overall survival analysis according to MALAT1 and BMI1 expression in subtypes of T and NK cell lymphomas using Kaplan-Meier survival curves.

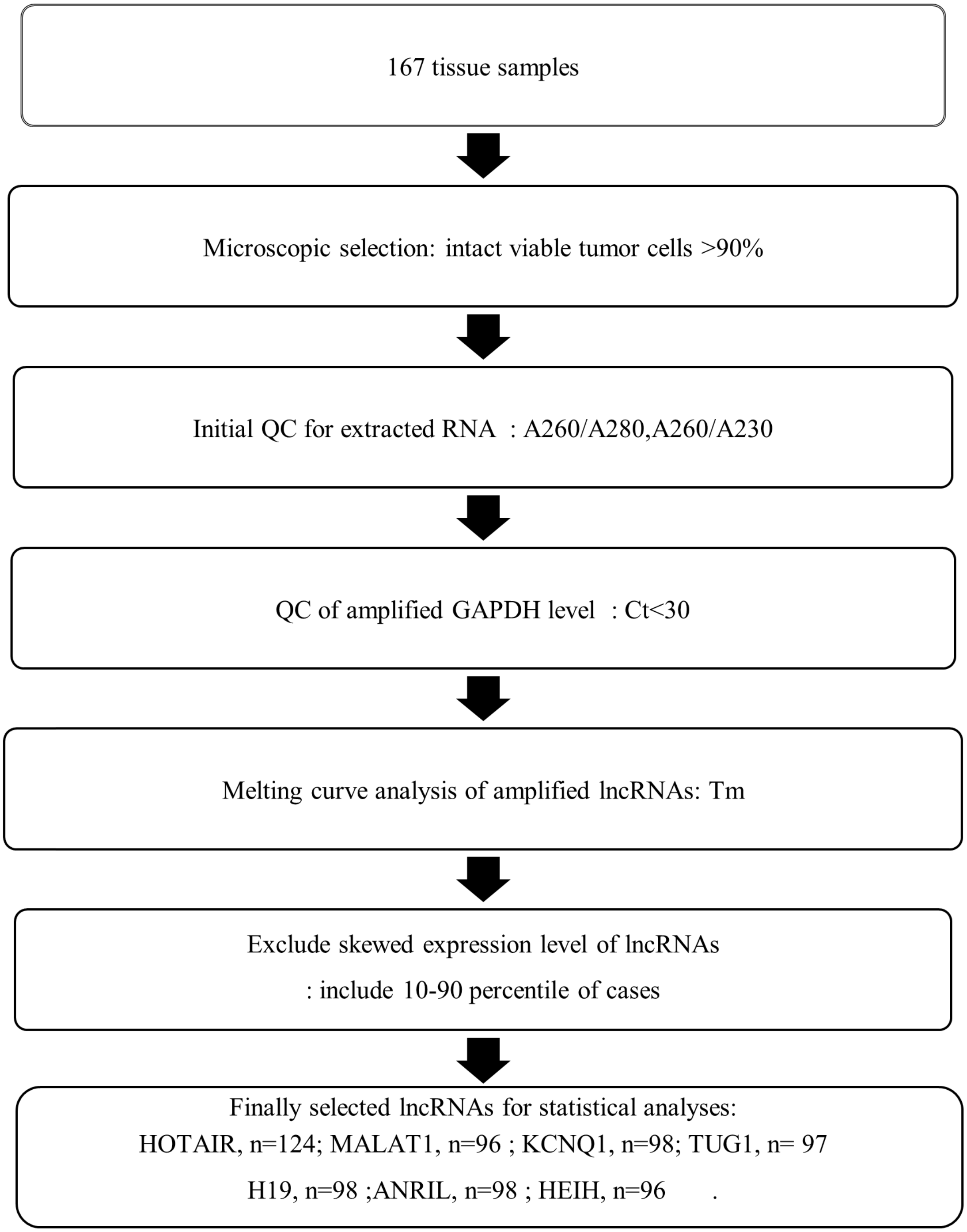

Supplementary Figure 4: Flow of case selection for lncRNA expression analysis.

Supplementary Table 1: Quantitative analysis of 7 long non-coding RNAs in clinical samples of T and NK cell lymphomas

	N	Median	Mean	SD
HOTAIR ratio	124	0.15	0.65	1.14
MALAT1 ratio	96	3.68	4.65	3.73
KCNQ1 ratio	98	0.02	0.16	0.31
TUG1 ratio	98	0.02	0.05	0.06
H19 ratio	97	0.09	0.34	0.65
ANRIL ratio	98	0.01	0.02	0.03
HEIH ratio	96	0.03	0.21	0.42

Supplementary Table 2: Characteristics of patients

Characteristic	n (%)
All patients	135
Sex	
Male	91(67.4%)
Female	44(32.6%)
Age (years)	
<60	86(63.7%)
≥60	49(36.3%)
Primary site of tumor	
Head and neck	36(26.7%)
Lymph node	72(53.3%)
Gastrointestinal tract	9(6.7%)
Soft tissue and bone	12(8.9%)
Others (solid organs)	6(4.4%)
Ann Arbor stage	
Stage I & II	28(20.7%)
Stage III&IV	75(55.6%)
Not evaluable	32(23.7%)
LDH level	
Normal	35(25.9%)
Elevated	60(44.4%)
Not evaluable	40(29.6%)
Bone marrow involvement	
Absent	72(53.3%)
Present	33(24.4%)
Not evaluable	30(22.2%)
IPI score	
0–2	61(45.2%)
3–5	43(31.9%)
Not evaluable	31(23.0%)

Abbreviations: LDH, lactate dehydrogenase; IPI, International Prognostic Index

Supplementary Table 3: Primers for seven types of lncRNAs, and the reference gene *GAPDH*

gene	primer-F
HOTAIR	AGCCAGAGGAGGGAAGAGAG
MALAT1	GCA GGG AGA ATT GCGTCA TT
KCNQ1	TCT CCC CAT GAT TTGCTG GT
TUG1	GTACCTCCA CTC AGCACA GT
H19	GAG GTT TAG GGGATC GAG GG
ANRIL	TTT ATT CCTGGCTCCCCT CG
HEIH	ACATAC CAG TGGCCAGAA GT
GAPDH	CAAATTCCATGGCACCGTCA
**Gene**	**primer-R**
HOTAIR	TCCCGTTCCCTAGATTTTCC
MALAT1	TTCTTCGCCTTCCGATAC TT
KCNQ1	TGAGAAAGGAAGGGG CAG AG
TUG1	CTG GAG TGGAGGGCTGTT AA
H19	TCTTGCTCTTTCTGCCTG GA
ANRIL	CGGAGCGGCTTT TAG TTC AA
HEIH	GTAGTCAGC CTC CCC TTC TG
GAPDH	ATCGCCCCACTTGATTTTGG

Supplementary Table 4: Cell lines and reagents

Cell lines	Origin	Media	Supplement
YT	EBV-positive human NK-like leukemia	Iscove's modified Dulbecco's medium (IMDM; 12440-046, Gibco; Life Technologies, CA, USA)	20% heat-inactivated fetal bovine serum (FBS; 6000-044, Gibco; Life Technologies)
SNK6	EBV-positive NKT cell lymphoma	RPMI1640 medium (22400-089, Gibco)	10% heat-inactivated human plasma and 700 U/mL of recombinant interleukin-2
HH	Cutaneous T cell lymphoma	RPMI1640 medium (22400-089, Gibco)	20% heat-inactivated FBS (6000-044, Gibco)
MAC1	Anaplastic lymphoma kinase (ALK)-negative anaplastic large cell lymphoma	RPMI1640 medium (22400-089, Gibco)	20% heat-inactivated FBS (6000-044, Gibco)
Jurkat	T lymphoblastic leukemia	RPMI1640 medium (22400-089, Gibco)	10% heat-inactivated FBS (6000-044, Gibco)
Toledo	Diffuse large B-cell lymphoma	in RPMI1640 media (22400-089, Gibco)	10% heat-inactivated FBS (6000-044, Gibco)

Supplementary Table 5: Sequences of si-MALAT1

si-MALAT1 type	Sequences
si-MALAT1-a	5'-CACAGAUGCUAUAGUACUA(dTdT)-3',<br>5'-UAGUACUAUAGCAUCUGUG(dTdT)-3'
si-MALAT1-b	5'-GUGGUAAACUAUACCUACU(dTdT)-3',<br>5'-AGUAGGUAUAGUUUACCAC(dTdT)-3'
si-MALAT1-c	5'-CAGAAGUGGAUUCAGUGAA(dTdT)-3',<br>5'-UUCACUGAAUCCACUUCUG(dTdT)-3'

Supplementary Table 6: The list of antibodies used for the RNA immunoprecipitation and western blot assays

Antibody or IgG	Clone or Cat No.	Manufacturer
EZH2	Rabbit monoclonal antibody D2C9 XP®	Cell Signaling Technology, MA, USA
SUZ12	Rabbit monoclonal antibody D39F6 XP®	Cell Signaling Technology, MA, USA
Tri-Methyl-Histone H3 (Lys27)	Rabbit monoclonal antibody C36B11	Cell Signaling Technology, MA, USA
BMI1	Rabbit monoclonal antibody D20B7XP®	Cell Signaling Technology, MA, USA
GAPDH	Rabbit monoclonal antibody 14C10	Cell Signaling Technology, MA, USA
Rabbit serum IgG	I5006	Sigma-Aldrich, MO, USA
Mouse serum IgG	I5381	Sigma-Aldrich, MO, USA
Anti-Rabbit IgG (whole molecule) antibody	R5506	Sigma-Aldrich, MO, USA
Anti-mouse IgG (whole molecule) antibody	M7023	Sigma-Aldrich, MO, USA

Supplementary Table 7: Antibodies used for immunohistochemistry

Antibody	Clone	Manufacturer	Dilution
EZH2	ZMD309	Invitrogen, Carlsbad, CA, USA	1:100
SUZ12	SUZ220A	Abcam, Cambridge, MA, USA	1:50
Tri-Methyl-HistoneH3 (Lys27)	C36B11	Cell Signaling Technology, Beverly, MA, USA	1:100
BMI1	1.T.21	Abcam, Cambridge, MA, USA	1:50
